# A novel mutation in *SPINK5* gene underlies a case of atypical Netherton syndrome

**DOI:** 10.3389/fgene.2022.943264

**Published:** 2022-09-09

**Authors:** Yu Wang, Hanqing Song, Lingling Yu, Nan Wu, Xiaodong Zheng, Bo Liang, Peiguang Wang

**Affiliations:** ^1^ Department of Dermatology, The First Affiliated Hospital, Anhui Medical University, Hefei, China; ^2^ Institute of Dermatology, Anhui Medical University, Hefei, China; ^3^ Key Laboratory of Dermatology, Anhui Medical University, Ministry of Education, Hefei, China; ^4^ Provincial Laboratory of Inflammatory and Immune Mediated Diseases, Hefei, China

**Keywords:** Netherton syndrome, *SPINK5*, congenital ichthyosiform erythroderma, mutation, phenotype

## Abstract

Netherton syndrome (NS, OMIM #256500) is a rare autosomal recessive disease characterized by a triad of congenital ichthyosiform erythroderma (CIE) or ichthyosis linearis circumflexa (ILC), trichorrhexis invaginata (TI), and atopic predisposition. The disease is caused by a mutation in the *SPINK5* gene (serine protease inhibitor of Kazal type 5) encoding LEKTI (lymphoepithelial Kazal type-related inhibitor). We performed whole-exome sequencing on one Chinese NS family and made genotype–phenotype correlation analysis on the patients clinically diagnosed with NS or congenital ichthyosis erythroderma. We identified a novel frameshift mutation c.2474_2475del (p.Glu825Glyfs*2) in the *SPINK5* gene. The N-terminal mutations of LEKTI cause a severer phenotype, while the C-terminal mutations of LEKT1 are related to a milder phenotype. Our findings suggest that Netherton syndrome may be underestimated clinically, and our findings further expand the reservoir of *SPINK5* mutations in Netherton syndrome.

## Introduction

Netherton syndrome (NS, OMIM #256500) was first described in 1958 by Earl Netherton. This disorder was characterized by a classical triad of congenital ichthyosiform erythroderma (CIE) or ichthyosis linearis circumflexa (ILC), trichorrhexis invaginata (TI), and atopic diathesis. The exact incidence of NS is unknown, but estimates range from 1:100,000 to 1:200,000 live births (Abdalrheem et al., 2020). Congenital ichthyosiform erythroderma is usually present at birth or shortly thereafter and manifests as generalized erythroderma with desquamation. Ichthyosis linearis circumflexa is characterized by migratory, serpiginous, erythematous patches with double-edged scales at the periphery. ILC is usually not permanent and can flare up after an interval (Hovnanian, 2013). The other common features of NS are recurrent infections, abnormal IgG level, bowel disease, hypernatremia, elevated IgE level, and low albuminemia (Śmigiel et al., 2017). NS is caused by a loss-of-function mutation of *SPINK5* (serine protease inhibitor of Kazal type 5) on chromosome 5q31-32 that encodes a lymphoepithelial Kazal-type related inhibitor (LEKTI) (Sarri et al., 2017; Herz-Ruelas et al., 2021). The LEKTI plays a critical role in maintaining skin barrier function and regulating the desquamation of keratinocytes (Chavanas et al., 2000; Flora and Smith, 2020).

Currently, there is no cure or satisfactory treatments for NS. Some modalities are used to relieve the disease, such as topical emollients, topical calcineurin inhibitors, narrowband ultraviolet B, psoralen plus UVA, topical or systemic corticosteroids, and topical or systemic acitretin (Lazaridou et al., 2009; Maatouk et al., 2012). Intravenous immunoglobulin and infliximab were attempted to treat a few NS patients with severe illness (Small and Cordoro, 2016; Roda et al., 2017).

Clinically, it is very difficult to distinguish atypical NS from congenital ichthyosiform erythroderma or other types of ichthyosis. Therefore, the mutation analysis of *SPINK5* gene is an important method in the diagnosis of atypical NS cases.

## Case presentation

### Ethical compliance

Following written informed consent, skin biopsies and hair samples were collected from the patient for histopathological examination and scanning electron microscope study at the Dermatology Clinic of The First Affiliated Hospital of Anhui Medical University, and blood samples were obtained from the patient and his family members for genetic analysis. This study was approved by the Ethics Committee of The First Affiliated Hospital of Anhui Medical University, and all procedures adhered to the principles of the Declaration of Helsinki.

### Report

**FIGURE 1:**
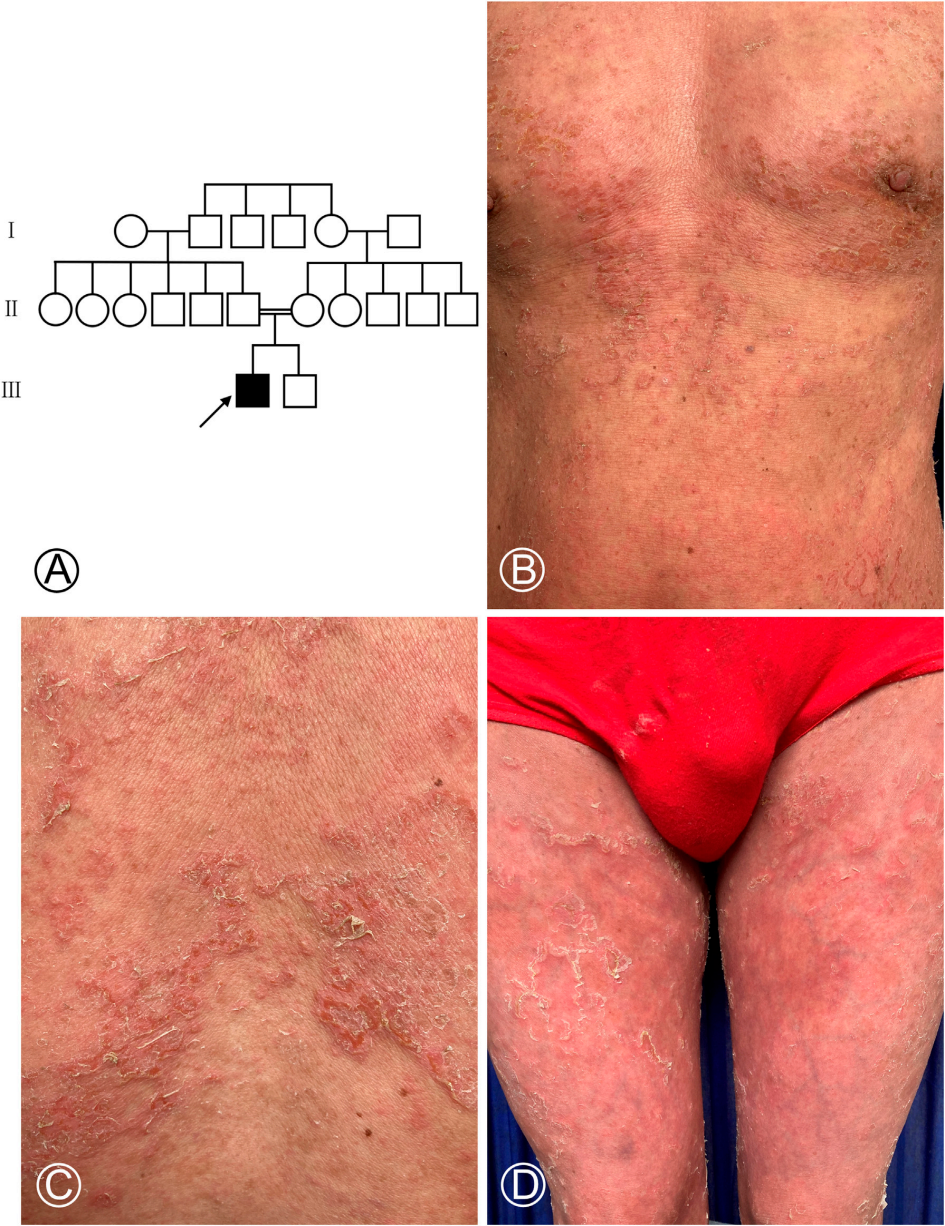
Pedigree chart of the NS-affected family and cutaneous manifestations of the proband. **(A)** Proband is marked with an arrow. Females were indicated by circles, while males were indicated by squares. The blackened symbol represented a diseased member. **(B–D)** Diffuse erythema covered with greyish white scales on the chest, abdomen, and both thighs and scattered multiple blisters in the chest.

**FIGURE 2:**
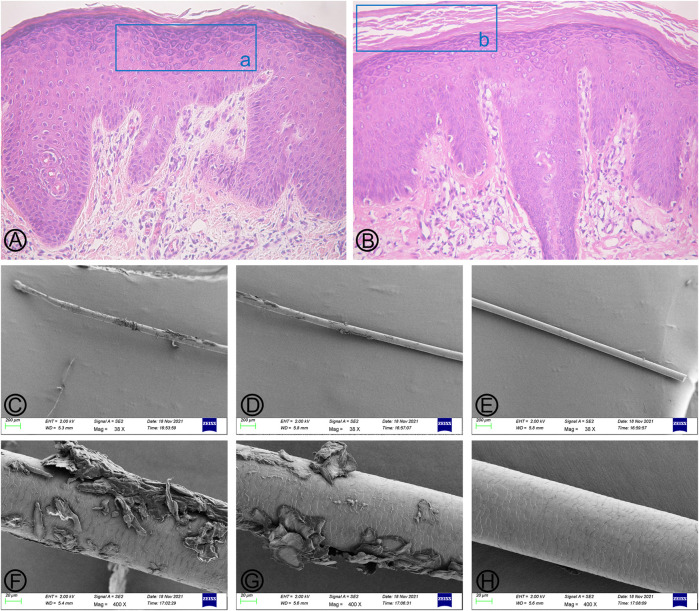
Findings of histopathological and hair scanning electron microscopy (SEM). **(A,B)** (HE×100) Marked hyperkeratosis, parakeratosis, hypergranulosis, and acanthosis, as well as dermal perivascular lymphocytic infiltrates. The blue box (a) indicates hypergranulosis, and the blue box (b) indicates hyperkeratosis. **(C–E)** (Mag×38); **(F–H)** (Mag×400) No characteristic bamboo hair was found under SEM. Some hair cuticles were damaged, deformed, and exfoliated in the two-thirds of the lower part of the hair shaft, while the hair cuticles were normal on the upper one-third of the hair shaft.

A 35-year-old male was admitted to our outpatient department for generalized erythema on his body with pruritus. The marriage of his parents was consanguineous. Some erythematous skin lesions were noted on his body 3 months after birth and gradually spread throughout the body with age. Sometimes, some blisters occurred. He denied a history of atopic dermatitis, asthma, or allergic rhinitis. Dermatological examination showed diffuse erythema with desquamation, and multiple flaccid vesicles over his whole body (Figure 1). His palms, soles, and oral mucosa were spared. His scalp hairs looked yellowish and lusterless and were easily plucked. His parents and younger brother lacked cutaneous abnormalities. However, his younger brother suffered from deafness since birth. Laboratory examination showed normal peripheral eosinophil counts and a significant increase in the serum IgE level with 749.95 IU/mL (normal range <200 IU/mL). Biochemical tests and serum levels of IgG, IgA, and IgM were all within normal limits. The histopathology of a biopsy revealed remarkable hyperkeratosis, parakeratosis, hypergranulosis, and acanthosis, as well as dermal perivascular lymphocytic infiltrates (Figure 2). Under the scanning electron microscope, his hairs showed the absence of trichorrhexis invaginata/trichorrhexis nodosa. Some hair cuticles were damaged, deformed, and exfoliated on two-thirds of the lower part of the hair shaft, while the hair cuticles were normal on the upper one-third of the hair shaft (Figure 2). Initially, we considered the diagnosis as bullous congenital ichthyosiform erythroderma (BCIE, OMIM #113800) based on the patient’s history and clinical and histopathological findings. None of the mutations in the related genes (*KRT1*, *KRT10*, and *KRT2e*) was found by Sanger sequencing in this family. Subsequently, whole-exome sequencing was performed to screen out a homozygous frameshift mutation in *SPINK5* gene, which was verified by Sanger sequencing. Although there was absence of trichorrhexis invaginata, the serum IgE level was significantly elevated, so this patient was finally diagnosed as affected by NS. According to the European guidelines for the management of congenital ichthyoses (Mazereeuw-Hautier et al., 2019), we recommended this patient be treated with oral acitretin, topical emollients, and topical corticosteroid ointment. Because the patient worried about adverse reactions to acitretin, he chose self-administered oral prednisone (10–15 mg/d) combined with topical emollients and topical corticosteroid ointment and maintained treatment for 6 months. The skin lesions on his chest and back improved significantly after 6 months of follow-up.

## Methods

### Peripheral blood collection and DNA extraction

After obtaining informed consent from all participants (including the proband, his parents, and his younger brother), EDTA-anticoagulated venous blood samples were collected from the patient and his family members for DNA analysis. DNA was extracted from peripheral blood with the use of the QIAamp DNA Mini kit (Qiagen, Valencia, CA, United States) according to the manufacturer’s instructions. The DNA concentration was measured using the Qubit 3.0 Fluorometer (Invitrogen, Life Technologies, Van Allen Way, Carlsbad, CA, United States), and DNA that met the criteria was selected and stored at −80°C.

### Mutation analysis of *KRT1*, *KRT10*, and *KRT2e* genes by Sanger sequencing

The mutations of *KRT1*, *KRT10*, and *KRT2e* genes in the proband, his parents, and his younger brother were analyzed by Sanger sequencing. Primers flanking all coding regions in *KRT1*, *KRT10*, *and KRT2e* were designed using Primer Premier 5.0 software (Primer Biosystems, Foster City, CA, United States). Primer sequences were provided in the supplementary data. The PCR system was configured, and the Veriti (ABI, Foster City, CA, United States) was set according to the standardized operation process, and the amplification was carried out according to the following conditions: 35 cycles of 30 s at 95°C; 30 s at 57°C; 120 s at 72°C. The reaction was ended with 20 min incubation at 72°C. PCR products were detected by 1.5% agarose gel electrophoresis. The Gel & PCR Clean-Up Kit D2000 (Omega Bio-Tek, Norcross, GA, United States) operation instructions were followed to purify and recycle the product. PCR products purified from genomic DNA were sequenced using an ABI 3730XL DNA Analyzer (ABI, Foster City, CA, United States). The sequencing results were analyzed using Finch TV (Version 1.5).

### Whole-exome sequencing

Since none of the mutations in *KRT1*, *KRT10*, or *KRT2e* genes was detected in the proband, his parents, or his younger brother, whole-exome sequencing (WES) was then performed on this family to screen for the deleterious mutation of the pathogenic gene. The patient and his younger brother were selected for WES. Genomic DNA was randomly broken into DNA fragments of 150–250 bp using a Covaris ultrasonic disintegrator (Covaris, Inc., Woburn, Massachusetts, United States). DNA libraries were prepared by repairing the ends and adding the Y-junction at both ends of the connecting fragment after the A-tail. Liquid phase hybridization was performed by pooling DNA libraries and whole-exome probes. All exons were captured through NimbleGen (Roche NimbleGen, Inc., Basel, Switzerland) with streptavidin-coated magnetic beads. The library quality was tested after linear PCR amplification, and then qualified libraries were used for high-throughput sequencing. High-throughput sequencing was performed on the Illumina HiseqXTen platform (Illumina, San Diego, CA, United States) using 2 × 100 bp paired-end (Diociaiuti et al., 2016; Nijman et al., 2014).

### Data analysis and interpretation

BWA-MEM was applied to compare the WES data with the HG19 version of the human genome. Quality control, variation comparison, variation identification, and annotation were performed on the raw data, and all possible pathogenic mutations were screened by comparison with allele frequency population databases such as ExAC database (http://exac.broad.insti.tute.org/dbsnp), 1000 Genomes database (http://www.inter.nationalge.nome.org/data-portal/sample), and KEGG database (https://www.kegg.jp/). The frequency of the identified variant in the Asian population has to be equal to zero or less than 1% in all the databases used. The identified variation was assessed by browsing databases including NCBI dbSNP (http://www.ncbi.nlm.nih.gov/SNP/), OMIM (http://www.omim.org/), HGMD (http://www.hgmd.cf.ac.uk/ac/index.php), and NCBI ClinVar (https://www.ncbi.nlm.nih.gov/ clinvar/). Combined with the phenotype of the proband, mutation interpretation was conducted according to the ACMG genetic variation classification criteria and guidelines.

### Verification by Sanger sequencing

**FIGURE 3:**
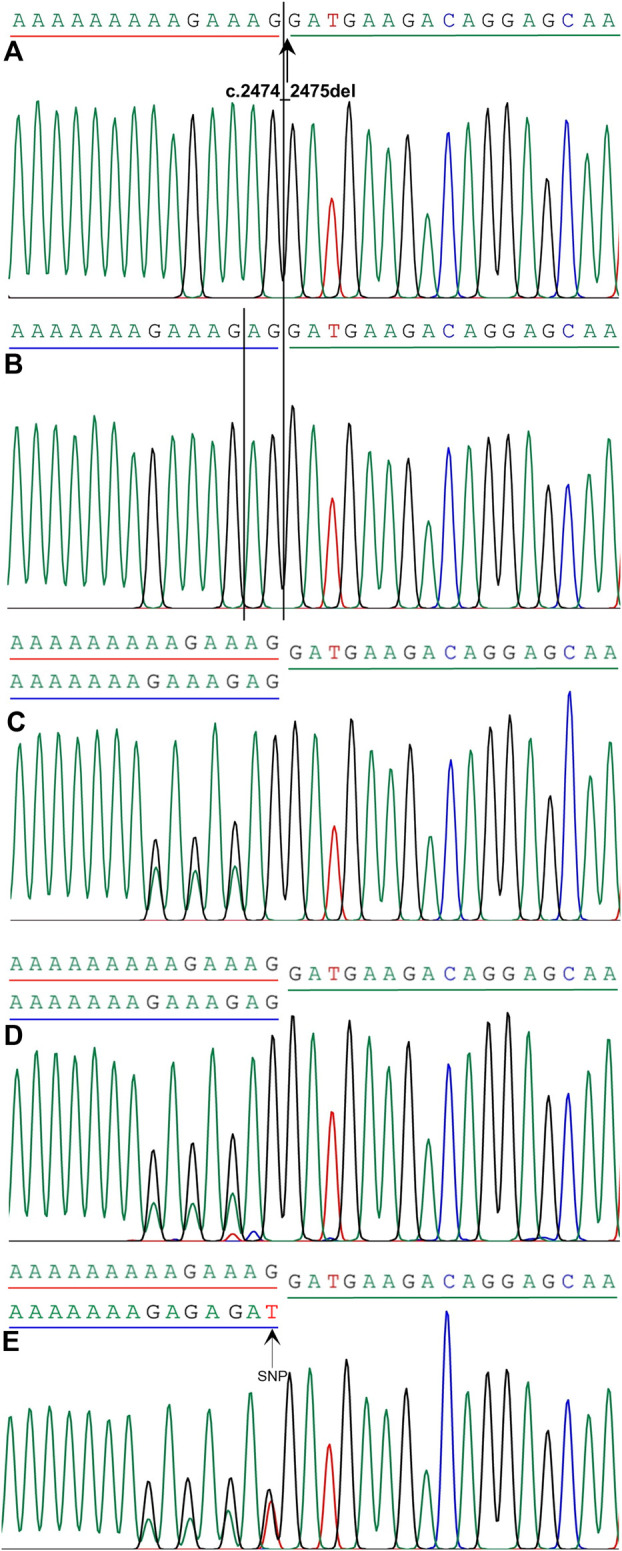
Mutation analysis of *SPINK5* gene in this family. **(A)** Homozygous frameshift mutation c.2474_2475del in *SPINK5* gene was identified in the proband. Reference sequencing of *SPINK5* gene in normal controls **(B)**, proband’s younger brother **(C)**, his father **(D)**, and his mother **(E)**. Also, single nucleotide polymorphism (SNP) in the DNA sequence of the proband’s mother was observed.

All reported mutations should be confirmed by Sanger sequencing, as false-positive results may occur with WES. When a mutation in the *SPINK5* gene was found by WES analysis, Sanger sequencing of *SPINK5* exons was performed in the proband, his parents, his younger brother, and 10 healthy controls independent of this family. PCR and Sanger sequencing of the *SPINK5* gene were performed using the aforementioned method to verify the genetic segregation pattern of this family. The sequences of primers used for validation sequencing were *SPINK5*-E25-26-Forward primer: GCC​TGA​CTC​TTG​GAA​AGA​AA and *SPINK5*-E25-26-Reverse primer: CAG​TTG​TCA​CTG​GTT​CTA​CA. The variation was identified by comparing with the reported cDNA reference sequences (GenBank Accession Number: NM_00112698, https://www.ncbi.nlm.nih.gov/genbank/). Also, Chromas (version 2.6.5) and PowerPoint were used to draw the sequence diagram of mutant genes (Figure 3).

### Clinical and genetic data collection

To further analyze the genotype–phenotype correlation of NS syndrome, we searched “Netherton syndrome” and “mutation” from the PubMed\Embase\MEDLINE\LILACS\Cochrane database and collected 91 relevant literature works in the last decade. By deleting duplicate literature works, removing review articles, and reading titles, abstracts, and full texts, 23 literature works with definite mutation sites in NS patients were obtained. A total of 25 NS patients were reported in detail. We summarized the clinical and genetic data of patients with Netherton syndrome.

## Results

### Mutation analysis of *KRT1*, *KRT10*, and *KRT2e* genes

The Sanger sequencing showed none of the mutations in *KRT1*, *KRT10*, *or KRT2e* gene in the patient, his younger brother, and his parents.

### WES and Sanger sequencing

We considered only substitutions, insertions, and deletions in the coding region, variants at canonical splicing sites in the noncoding region, and variations at canonical splicing sites, excluding variations with a minor allele frequency greater than 0.01 in different common and local resources. A homozygous frameshift mutation in the *SPINK5* gene (NM_001127698), c.2474_2475del (p.Glu825Glyfs*2) in exon 26, was identified after integrative analysis (Figure 3). The mutation was not present in ExAC, 1000 Genomes databases, KEGG databases, and others and leads to a frameshift and a premature stop codon. Sanger sequencing showed that this variation was not observed in other family members with normal skin phenotypes or in 10 healthy controls independent of this family. His parents and his younger brother were the heterozygous carriers of this mutation (Figure 3).

### Clinical and genetic data analysis

The clinical and genetic data of 25 individuals with NS were summarized (Table 1). Only 12 (48%) patients exhibited the classical triad of congenital ichthyosiform erythroderma (CIE) or ichthyosis linearis circumflexa (ILC), trichorrhexis invaginata (TI), and atopic diathesis. All patients showed scaly erythroderma. However, this condition was absent at birth in six patients. About half of the patients were presented with ILC. TI, atopic diathesis, and high IgE levels were observed in 20 (80%), 15 (60%), and 13 (52%) patients, respectively. Two patients died of skin barrier destruction and septicemia among 25 NS patients. However, their IgE levels were normal or mildly elevated, suggesting that the IgE levels are not correlated with the severity of the disease.

**TABLE 1:** Summary of the clinical phenotype and genotype from patients with Netherton syndrome.

	Age^1^	Sex	SE	Diagnostic parameters				LEKTI detection	SPINK5 mutation				Treatment
				ILE/CIE	TI	Atopic manifestation	Other systemic finding		Location (exon)	Pathogenic variant	Nucleotide change	Reference	
1	3 years	M	+	ILC	+	E, EO, and H-IgE: 35,200 U/ml	−	NM	Exon 2	c.80A > G	p.Gln27Arg	Zhang et al. (2021)	IVIG therapy
2	1 month	ND	+	CIE	ND	−	HN	NM	Exon 3	c.153delT	p.Gln52LysfsTer6	Tiryakioğlu et al. (2017)	Specially prepared ointments
3	13 years	M	+	CIE	+	NM	NM	NM	Exon 4	c.238_239insG	p.Ala80fs	Schepis et al. (2019)	NM
4	20 years	F	+	CIE	+	NM	NM	NM	Exon 4	c.238_239insG	p.Ala80fs	Schepis et al. (2019)	NM
5	23 years	F	+	ILC	+	A, AR, FA, and H-IgE: 1,373 U/ml	−	NM	Exon 5	c.316_317delGA	p.Asp106TrpfsTer7	Kogut et al. (2015)	Topical calcineurin inhibitors and moisturizers
									Exon 26	c.2468dupA	p.Les824GlufsTer3		
6	18 years	F	+	ILC	+	H-IgE: 2,176 U/ml	−	Negative	Exon 5	c.318G > A	p.Asp106Ter	Xi-Bao et al. (2012)	NM
7	6 months	F	+	CIE	+	FA and H-IgE: 708 U/ml	HN	NM	Exon 5	c.377_378delAT	p.Tyr126Ter	Konishi et al. (2014)	NM
									Exon 25	c.2368C > T	p.Arg790Ter		
8	2 years	M	+	ILC	+	H-IgE: 528 U/ml	−	Negative	Exon 6	c.474G > A	p.Gln158 =	Numata et al. (2016)	NM
									Exon 19	c.1732C > T	p.Arg578Ter		
9	6 years	F	-	CIE	+	FA	GR	NM	Exon 7	c.581_82delGT	p.Cys194fsTer4	Aydın et al. (2014)	Growth hormone therapy
10	22 years	M	−	ILC	+	FA and H-IgE: 1,878 U/ml	−	NM	Exon 8	c.649C > T	p.Arg217Ter	Alpigiani et al. (2012)	NM
									Exon 11	c.957_960dupTGGT	p.Pro321TrpfsTer23		
11	10 months	M	+	CIE	+	H-IgE: 951 U/ml	GR, HP, and IN	Negative	Exon 8	c.652C > T	p.Arg218Ter	Wang et al. (2021)	IVIG therapy, topical emollients, corticosteroid cream, and antibiotic cream
12	11 months	F	−	CIE	+	−	GR, HN, IA, and IN	NM	Exon 11	c.995delT	p.Met332SerfsX43	Nevet et al. (2017)	Acitretin 1 mg/kg and topical humectants (death)
13	10 years	M	+	ILC	+	E and H-IgE: >10,000U/ml	IN	NM	Exon 11	c.997C > T	p.Gln333Ter	Fong et al. (2011)	Topical emollients and corticosteroid cream
14	5 years	F	−	ILC	+	−	IN	NM	Exon 13	c.1099dupT	p.Cys367Leufs*3	Konishi et al. (2014)	NM
									Exon 25	c.2368C > T	p.Arg790Ter		
15	6 months	M	+	CIE	+	−	AN, HN, HP, IN, and SZ	Negative	Exon 13	c.1111C > T	p.Arg371X	Diociaiuti et al. (2013)	(Death)
16	2 years	M	−	ILC	−	NM	NM	Negative	Exon 15	c.1258G > A	p.Glu420Lys	Guerra et al. (2015)	Topical steroid
									Intron 14	c.1302+4A > T			
17	15 years	M	+	CIE	ND	H-IgE: 10,700 U/ml	ALL and IN	NM	Exon 17	c.1530C > A	p.Cys510Ter	Skoczen et al. (2020)	Chemotherapy, topical emollients, and corticosteroid cream
18	2 years	M	+	ILC	+	−	GR, HN, HO, HP, and IN	NM	Exon 17	c.1530C > A	p.Cys510Ter	Zelieskova et al. (2020)	IVIG therapy
19	50 months	M	+	ILC	+	AO and H-IgE: 11,425 U/ml	−	Negative	Exon 19	c.1772delT	p.Leu591GlnfsTer124	Hannula-Jouppi et al. (2016)	Acitretin treatment showed no benefit
20	42 years	M	+	ILC	ND	E	IN and IVL	Negative	Exon 26	c.2468dupA	p.Les824GlufsTer3	Albluwi et al. (2020)	Topical emollients, antibiotic cream, and oral antibiotics
									Intron 4	c.283-2A > T			
21	6 moths	M	−	ILC	+	E, EO, and H-IgE: 453 U/ml	−	NM	Intron 5	c.410 +1G > A		Cicek et al. (2021)	Infliximab therapy
22	8 months	M	+	CIE	+	−	BH, HN, HO, and IN	NM	Intron 15	c.1431-12G > A		Śmigiel et al. (2017)	NM
									Intron 19	c.1816-1820 + 21delinsCT			
23	5 months	M	+	CIE	+	−	HN and HO	NM	Intron 17	c.1608-1G>a		Okulu et al. (2018)	ECMO support, topical Aquaphor, and bronchodilators
24	20 years	M	+	CIE	+	H-IgE: >2,000 U/ml	−	NM	Intron 17	c.1608-1G>a		Erden et al. (2020)	NM
25	4 years	M	+	ILC	−	E, AO, and H-IgE: 3,420 U/ml	−	NM	Intron 22	c.2112+2T > A		Özyurt et al. (2019)	Acitretin 0.5 mg/kg and topical moisturizers

Abbreviations: A, asthma; ALL, acute lymphoblastic leukemia; AN, anemia; AO, angioedema; AR, allergic rhinitis; BH, bilateral hypoacusia; CIE, congenital ichthyosiform erythroderma; E, eczematous-like rashes; ECMO, extracorporeal membrane oxygenation; EO, eosinophilia; F, female; FA, food allergy; GR, growth retardation; H-IgE, hyper-IgE; HN, hypernatronemia; HO, hypoxemia; HP, hypoproteinemia; IA, intestinal atresia; ILC, ichthyosis linearis circumflexa; IN, infection; IVIG, intravenous immunoglobulin; IVL, inflammatory vegetative lesions; M, male; NM, not mentioned; SE, scaly erythroderma at birth; SZ, seizure; TI, trichorrhexis invaginata.

### Genotype–phenotype correlation analysis

So far, more than 80 different mutations in the *SPINK5* gene have been reported. Most of the mutations in *SPINK5* responsible for the NS phenotype introduce a premature termination codon (PTC), resulting in a lack of detectable LEKTI expression (Zelieskova et al., 2020; Hovnanian, 2013). The phenotypes of NS were variable with manifestations from mild clinical signs to life-threatening complications, especially during the neonatal period. Some patients with truncated mutations located early in the coding sequence have a more severe phenotype, while the mutations in *SPINK5* gene that interrupt coding frames near the C-terminus may allow the retention of functional LEKTI fragments, leading to a milder phenotype.

## Discussion

Netherton syndrome is a rare autosomal recessive disease with skin, hair, and immune abnormalities. The anomaly of the hair shaft is highly specific in NS patients, but the absence of this condition does not rule out the diagnosis of NS (Torchia and Schachner, 2011). The scalp hairs, eyebrows, or eyelashes may be lusterless, sparse, and brittle. Light microscopy examination almost always shows characteristic bamboo hair or trichorrhexis invaginata (TI), which refers to the cup-shaped protrusion of the distal part of the hair shaft toward the proximal part (Hovnanian, 2013). Hair examination is sometimes difficult because not every hair is affected. The eyebrows are considered a prior choice to visualize this abnormality (Bittencourt et al., 2015). In addition, a few patients with NS only have ichthyosis linearis circumflexa (ILC) but do not have the changes of trichorrhexis invaginata (TI) (Guerra et al., 2015; Özyurt et al., 2019).

Two-thirds of NS patients suffer from allergic diseases including atopic dermatitis, allergic asthma, allergic rhinitis, anaphylactic reactions to food, urticaria, and angioedema, as well as elevated serum IgE level and blood hypereosinophilia (Erden et al., 2020). Some severe comorbidities were also seen in a minority of NS neonates, such as skin infection, enteropathy, hypernatremia, hypoproteinemia, sepsis, and growth retardation (Hernández-Martín and González-Sarmiento, 2015).

The diagnosis of NS is straightforward in the presence of characteristic cutaneous manifestations, trichorrhexis invaginata, atopic diathesis, and a positive family history (Leung et al., 2018). However, it is still challenging to make an accurate diagnosis for the NS cases that lack a typical clinical phenotype. Some powerful diagnostic tests have facilitated the recognition of these cases of NS, such as LEKTI immunodetection in the skin and mutation analysis of *SPINK5* gene (Lacroix et al., 2012).

The *SPINK5* gene contains 33 exons spanning 61 kb and encodes LEKTI expressing in the skin, mucosa, and thymic epithelium (Hovnanian, 2013). The pathogenic mutations in the *SPINK5* gene result in a truncation of LEKTI, which reduce LEKTI’s ability to inhibit skin serine proteases (Kasparek et al., 2017; Tiryakioğlu et al., 2017). The deficiency of LEKTI leads to hyperactivation of skin kallikreins (KLK) and increased degradation of desmosine and corneodesmosomal cadherins. Consequently, the defect of the skin barrier occurs following abnormal skin homeostasis and detachment of the stratum corneum. KLK5 can activate protease-activated receptor 2 (PAR-2) and lead to increased expression of thymic stromal lymphopoietin (TSLP), TNF-α, IL-8, and ICAM-1 through the KLK5-PAR2 cascade, thereby enhancing the inflammatory process (Stuvel et al., 2020). The loss of LEKTI expression in the trachea can lead to the destruction of the airway epithelial barrier, further making NS patients more susceptible to inhaled allergens (Berthold et al., 2016). LEKTI deficiency leads to increased cleavage of cross-linkage in hair keratin structures, local defect of the inner root sheath, and morphological change of trichorrhexis invaginata (Bittencourt et al., 2015). The decreased expression of LEKTI in the sinonasal epithelium is associated with allergic rhinitis (Hannula-Jouppi et al., 2014).

To date, more than 80 kinds of mutations have been identified in intronic and exonic regions of the *SPINK5* gene. Most of the *SPINK5* mutations causing NS introduce a premature termination codon (PTC) which results in a lack of detectable LEKTI expression (Furio et al., 2014). The primary structure of LEKTI consists of 15 serine protease inhibitory domains (Śmigiel et al., 2017), in which the D6–D9 domains of LEKTI possess the most effective inhibiting activity on KLK (Guerra et al., 2015). A more severe phenotype was observed in some patients with truncated mutations located early in the coding sequence, which may be due to a substantial reduction or complete lack of LEKTI expression (Diociaiuti et al., 2013; Nevet et al., 2017), whereas a milder phenotype was related to the *SPINK5* mutations interrupting coding frames near the C-terminus, which allow the retention of functional LEKTI fragments (Guerra et al., 2015). In addition, some deep intronic mutations activate the hidden splicing sites and cause a milder phenotype (Śmigiel et al., 2017).

**FIGURE 4:**
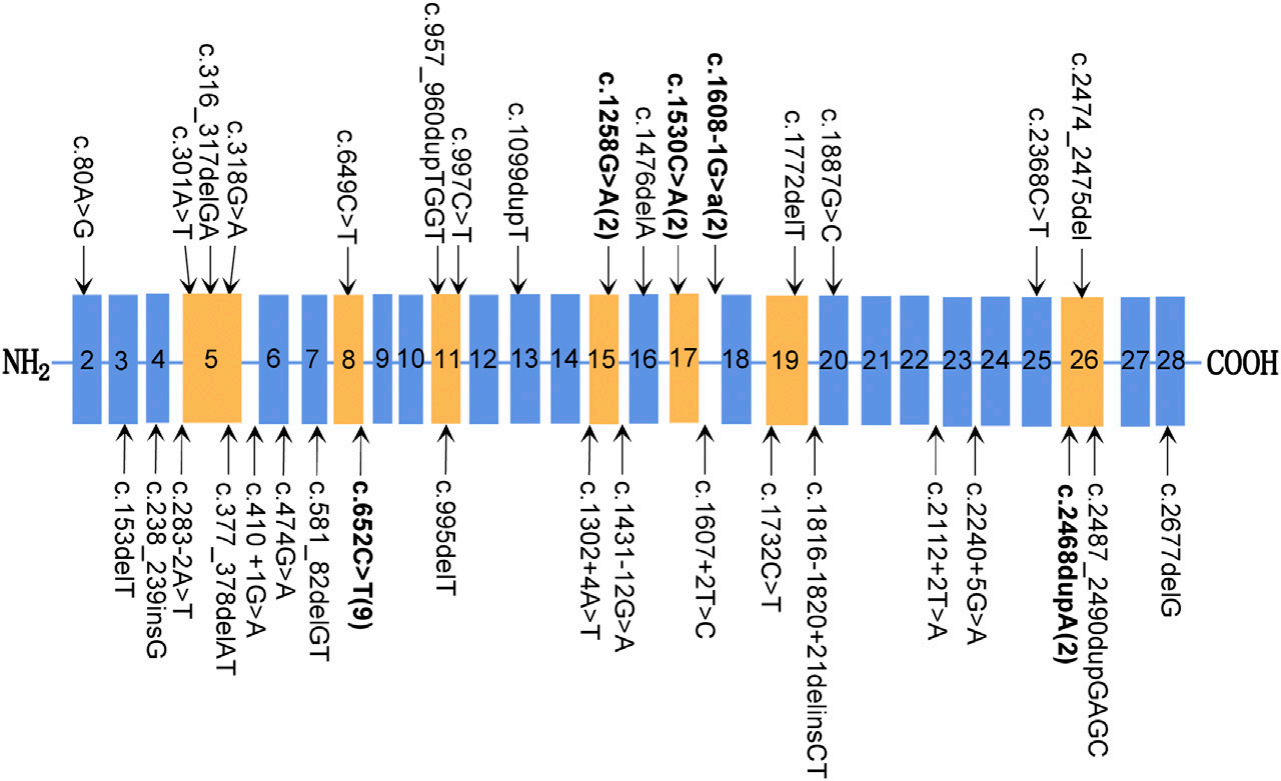
Mutations of the *SPINK5* gene. Typical exon domains are represented in orange, and other exon domains are depicted in blue. Mutations occurring more than once are shown in bold, and the number of unrelated families carrying this mutation is shown in parentheses.

Genotype–phenotype correlation analysis shows that more severe phenotypes were associated with the mutations of exons 1–25 in the *SPINK5* gene (Table 1; Figure 4). None of the mutations in the downstream of exon 28 was detected in the NS patients. Mostly, the mutations of exon 26 were compound heterozygous mutations [c.283-2A > T in intron 4 and c.2468dupA in exon 26 (Albluwi et al., 2020); c.316_317delGA in exon 5 and c.2468dupA in exon 26 (Kogut et al., 2015); and c.2260A > T in exon 24 and c.2468delA in exon 26 (Chao et al., 2003)]. In our study, the patient carried a homozygous mutation c.2474_2475del on exon 26 and displayed a milder phenotype, which is probably related to the mutation site near the C-terminal of LEKTI. The number of mutations in exons 1–17 of *SPINK5* gene was significantly more than that in exons 18–33 (Figure 4), which may be related to neglecting of those patients with the milder phenotype. The prevalence of NS is likely underestimated. Therefore, it is recommended to make gene panel (*KRT1*, *KRT10*, *KRT2e*, and *SPINK5*) testing for those patients with congenital ichthyosiform erythroderma (CIE) or ichthyosis linearis circumflexa (ILC). The method is beneficial to improve the diagnosis of NS. The diagnosis of this patient was demonstrated by mutation analysis of the *SPINK5* gene.

In conclusion, we describe a case of Netherton syndrome without the appearance of trichorrhexis invaginata and a novel homozygous *SPINK5* frameshift mutation. Our findings further expand the spectrum of both clinical phenotypes of NS and mutations of *SPINK5* gene.

## Data Availability

The datasets for this article are not publicly available due to concerns regarding participant/patient anonymity. Requests to access the datasets should be directed to the corresponding author.
