# Supplementary material for: A novel mutation in SPINK5 gene underlies a case of atypical Netherton syndrome
Source: Front Genet. 2022 Sep 9;13:943264. doi: 10.3389/fgene.2022.943264 (PMC9500337; doi:10.3389/fgene.2022.943264)
Supplement: Supplementary file 1 [file DataSheet2.PDF]

SPINK5: NM_001127698: exon26: c.2474_2475del: p.Glu825Glyfs*2

tctctgtgggacattattttgcctatcacagcaaggttacatggctgcctgactcttggaaagaaatcctctgattc
tcaaatccaatcaaatattatgtaaaaacagcacttccaatataatcttcccatcttttcaggatacatgtgatgag
tttagaagccaaatgaaaaatggaaaactcatctgcactcgagaaagtgaccctgtccggggtccagatggcaagac
acatggcaataagtgtactatgtgtaaggaaaaactgtgagtatgtttcaaaatgagcttttgactgtgagtcttaa
agtacaataatcatttcttaccagtttgggaaaatgacaattgttttagaagcagatctggtaattaatgaggcgtt
tgttcactttgattgaaatgtttcattgttttccccccagggaaagggaagcagctgaaaaaaaaaagaaagaggat
gaagacaggagcaatacaggagaaaggagcaatacaggagaaaggagcaatgacaaagaggtaatagatgttagaca
cgctaatacctgaattcagttagttcattgtatggtatatttattcaacaaatatttgtgaaatgctgactctgtcc
caatcattggtgatataacggtaaacaatgaagtcatggccagatcttgaaaataaatagcatgctcttcagttccc
caggagtgactctgatgcaattgtagaaccagtgacaactgtcaaattattgtagttagccagtgaatttcattttt

批注 [王1]: Del
