# Supplementary material for: A novel mutation in SPINK5 gene underlies a case of atypical Netherton syndrome
Source: Front Genet. 2022 Sep 9;13:943264. doi: 10.3389/fgene.2022.943264 (PMC9500337; doi:10.3389/fgene.2022.943264)
Supplement: Supplementary file 2 [file DataSheet3.PDF]

NM_001127698 3741 bp mRNA
ORIGIN

```
    1 agtcatactg caccagctga gcaatgcatg gagtggacct gtaggcgact tgcatcgtct
   61 tcaacatgaa gatagccaca gtgtcagtgc ttctgccctt ggctctttgc ctcatacaag
  121 atgctgccag taagaatgaa gatcaggaaa tgtgccatga atttcaggca tttatgaaaa
  181 atggaaaact gttctgtccc caggataaga aattttttca aagtcttgat ggaataatgt
  241 tcatcaataa atgtgccacg tgcaaaatga tactggaaaa agaagcaaaa tcacagaaga
  301 gggccaggca tttagcaaga gctcccaagg ctactgcccc aacagagctg aattgtgatg
  361 attttaaaaa aggagaaaga gatggggatt ttatctgtcc tgattattat gaagctgttt
  421 gtggcacaga tgggaaaaca tatgacaaca gatgtgcact gtgtgctgag aatgcgaaaa
  481 ccgggtccca aattggtgta aaaagtgaag gggaatgtaa gagcagtaat ccagagcagg
  541 atgtatgcag tgcttttcgg ccctttgtta gagatggaag acttggatgc acaagggaaa
  601 atgatcctgt tcttggtcct gatgggaaga cgcatggcaa taagtgtgca atgtgtgctg
  661 agctgttttt aaaagaagct gaaaatgcca agcgagaggg tgaaactaga attcgacgaa
  721 atgctgaaaa ggatttttgc aaggaatatg aaaaacaagt gagaaatgga aggctttttt
  781 gtacacggga gagtgatcca gtccgtggcc ctgacggcag gatgcatggc aacaaatgtg
  841 ccctgtgtgc tgaaattttc aagcagcgtt tttcagagga aaacagtaaa acagatcaaa
  901 atttgggaaa agctgaagaa aaaactaaag ttaaaagaga aattgtgaaa ctctgcagtc
  961 aatatcaaaa tcaggcaaag aatggaatac ttttctgtac cagagaaaat gaccctattc
 1021 gtggtccaga tgggaaaatg catggcaact tgtgttccat gtgtcaagcc tacttccaag
 1081 cagaaaatga agaaaagaaa aaggctgaag cacgagctag aaacaaaaga gaatctggaa
 1141 aagcaacctc atatgcagag ctttgcagtg aatatcgaaa gcttgtgagg aacggaaaac
 1201 ttgcttgcac cagagagaac gatcctatcc agggcccaga tgggaaagtg catggcaaca
 1261 cctgctccat gtgtgaggtc ttcttccaag cagaagaaga agaaaagaaa aagaaggaag
 1321 gtaaatcaag aaacaaaaga caatctaaga gtacagcttc ctttgaggag ttgtgtagtg
 1381 aataccgcaa atccaggaaa aacggacggc tttttgcac cagagagaat gaccccatcc
 1441 agggcccaga tggaaaaatg catggcaaca cctgctccat gtgtgaggcc ttctttcaac
 1501 aagaagaaag agcaagagca aaggctaaaa gagaagctgc aaaggaaatc tgcagtgaat
 1561 ttcgggacca agtgaggaat ggaacactta tatgcaccag ggagcataat cctgtccgtg
 1621 gcccagatgg caaaatgcat ggaaacaagt gtgccatgtg tgccagtgtg ttcaaacttg
 1681 aagaagaaga gaagaaaaat gataaagaag aaaaagggaa agtcgaggct gaaaaagtta
 1741 agagagaagc agttcaggag ctgtgcagtg aatatcgtca ttatgtgagg aatggacgac
 1801 tcccctgtac cagagagaat gatcctattg agggtctaga tgggaaaatc cacggcaaca
 1861 cctgctccat gtgtgaagcc ttcttccagc aagaagcaaa agaaaaagaa agagctgaac
 1921 ccagagcaaa agtcaaaaga gaagctgaaa aggagacatg cgatgaattt cggagacttt
 1981 tgcaaaatgg aaaacttttc tgcacaagag aaaatgatcc tgtgcgtggc ccagatggca
 2041 agacccatgg caacaagtgt gccatgtgta aggcagtctt ccagaaagaa aatgaggaaa
 2101 gaaagaggaa agaagaggaa gatcagagaa atgctgcagg acatggttcc agtggtggtg
 2161 gaggaggaaa cactcaggac gaatgtgctg agtatcggga acaaatgaaa aatggaagac
 2221 tcagctgtac tcgggagagt gatcctgtac gtgatgctga tggcaaatcg tacaacaatc
 2281 agtgtaccat gtgtaaagca aaattggaaa gagaagcaga gagaaaaaat gagtattctc
 2341 gctccagatc aaatgggact ggatcagaat cagggaagga tacatgtgat gagtttagaa
 2401 gccaaatgaa aaatggaaaa ctcatctgca ctcgagaaag tgaccctgtc cggggtccag
 2461 atggcaagac acatggcaat aagtgtacta tgtgtaagga aaaactggaa agggaagcag
```

2521 ctgaaaaaaa aaagaaagag gatgaagaca ggagcaatac aggagaaagg agcaatacag
2581 gagaaaggag caatgacaaa gaggatctgt gtcgtgaatt tcgaagcatg cagagaaatg
2641 gaaagcttat ctgcaccaga gaaaataacc ctgttcgagg cccatatggc aagatgcaca
2701 tcaataaatg tgctatgtgt cagagcatct ttgatcgaga agctaatgaa agaaaaaaga
2761 aagatgaaga gaaatcaagt agcaagccct caaataatgc aaaggaccag tgcagacagg
2821 ttcagaatga agcggaggat gcaaaattta gacaacctgg gcgttccttg gcctctgttg
2881 ccaggatgag tacagatgag tgcagtgaat ttcgaaacta tataaggaac aatgaactca
2941 tctgccctag agagaatgac ccagtgcacg gtgctgatgg aaagttctat acaaacaagt
3001 gctacatgtg cagagctgtc tttctaacag aagctttgga aagggcaaag cttcaagaaa
3061 agccatccca tgttagagct tctcaagagg aagacagccc agactctttc agttctctgg
3121 attctgagat gtgcaaagac taccgagtat tgcccaggat aggttatctt tgtccaaagg
3181 atttaaagcc tgtctgtggt gacgatggcc aaacctacaa caatccttgc atgctctgtc
3241 atgaaaacct gatacgccaa acaaatacac acatccgcag tacagggaag tgtgaggaga
3301 gcagcacccc aggaaccacc gcagccagca tgcccccgtc tgacgaatga caggaagatt
3361 gttgaaagcc atgagggaaa aaataaaccc cagttctgaa tcacctacct tcaccatctg
3421 tatatacaaa gaattcttcg gagcttgtct tatttgctat agaaaacaat acagagcttt
3481 tgggaatgga ctcactgatt ttcagtcttt tccatctctt tcctcctaga ctctgtgatc
3541 tgagggtata aagacatctc caccaagtct gagccctcaa aatgtcctga ttacaatgct
3601 gtctgtccaa ctgcctgttc aataaaagta aactcagcag aacacccttt ctgggatttc
3661 tttgtcacta tctggataat agatatttgc ttttaaagaa actgaataaa ctctaccctt
3721 ttgtcttttt gtgttgctaa a

NM_006846 3651 bp mRNA ENST00000256084.8

ORIGIN

1 agtcatactg caccagctga gcaatgcatg gagtggacct gtaggcgact tgcatcgtct
61 tcaacatgaa gatagccaca gtgtcagtgc ttctgccctt ggctctttgc ctcatacaag
121 atgctgccag taagaatgaa gatcaggaaa tgtgccatga atttcaggca tttatgaaaa
181 atggaaaact gttctgtccc caggataaga aattttttca aagtcttgat ggaataatgt
241 tcatcaataa atgtgccacg tgcaaaatga tactggaaaa agaagcaaaa tcacagaaga
301 gggccaggca tttagcaaga gctcccaagg ctactgcccc aacagagctg aattgtgatg
361 attttaaaaa aggagaaaga gatggggatt ttatctgtcc tgattattat gaagctgttt
421 gtggcacaga tgggaaaaca tatgacaaca gatgtgcact gtgtgctgag aatgcgaaaa
481 ccgggtccca aattggtgta aaaagtgaag gggaatgtaa gagcagtaat ccagagcagg
541 atgtatgcag tgcttttcgg ccctttgtta gagatggaag acttggatgc acaagggaaa
601 atgatcctgt tcttggtcct gatgggaaga cgcatggcaa taagtgtgca atgtgtgctg
661 agctgttttt aaaagaagct gaaaatgcca agcgagaggg tgaaactaga attcgacgaa
721 atgctgaaaa ggatttttgc aaggaatatg aaaaacaagt gagaaatgga aggctttttt
781 gtacacggga gagtgatcca gtccgtggcc ctgacggcag gatgcatggc aacaaatgtg
841 ccctgtgtgc tgaaattttc aagcagcgtt tttcagagga aaacagtaaa acagatcaaa
901 atttgggaaa agctgaagaa aaaactaaag ttaaaagaga aattgtgaaa ctctgcagtc
961 aatatcaaaa tcaggcaaag aatggaatac ttttctgtac cagagaaaat gaccctattc
1021 gtggtccaga tgggaaaatg catggcaact tgtgttccat gtgtcaagcc tacttccaag
1081 cagaaaatga agaaaagaaa aaggctgaag cacgagctag aaacaaaaga gaatctggaa
1141 aagcaacctc atatgcagag ctttgcagtg aatatcgaaa gcttgtgagg aacggaaaac

1201 ttgcttgcac cagagagaac gatcctatcc agggcccaga tgggaaagtg catggcaaca
1261 cctgctccat gtgtgaggtc ttcttccaag cagaagaaga agaaaagaaa aagaaggaag
1321 gtaaatcaag aaacaaaaga caatctaaga gtacagcttc ctttgaggag ttgtgtagtg
1381 aataccgcaa atccaggaaa aacggacggc ttttttgcac cagagagaat gacccccatcc
1441 agggcccaga tggaaaaatg catggcaaca cctgctccat gtgtgaggcc ttctttcaac
1501 aagaagaaag agcaagagca aaggctaaaa gagaagctgc aaaggaaatc tgcagtgaat
1561 ttcgggacca agtgaggaat ggaacactta tatgcaccag ggagcataat cctgtccgtg
1621 gcccagatgg caaaatgcat ggaaacaagt gtgccatgtg tgccagtgtg ttcaaacttg
1681 aagaagaaga gaagaaaaat gataaagaag aaaaagggaa agtcgaggct gaaaaagtta
1741 agagagaagc agttcaggag ctgtgcagtg aatatcgtca ttatgtgagg aatggacgac
1801 tcccctgtac cagagagaat gatcctattg agggtctaga tgggaaaatc cacggcaaca
1861 cctgctccat gtgtgaagcc ttcttccagc aagaagcaaa agaaaaagaa agagctgaac
1921 ccagagcaaa agtcaaaaga gaagctgaaa aggagacatg cgatgaattt cggagacttt
1981 tgcaaaatgg aaaacttttc tgcacaagag aaaatgatcc tgtgcgtggc ccagatggca
2041 agacccatgg caacaagtgt gccatgtgta aggcagtctt ccagaaagaa aatgaggaaa
2101 gaaagaggaa agaagaggaa gatcagagaa atgctgcagg acatggttcc agtggtggtg
2161 gaggaggaaa cactcaggac gaatgtgctg agtatcggga acaaatgaaa aatggaagac
2221 tcagctgtac tcgggagagt gatcctgtac gtgatgctga tggcaaatcg tacaacaatc
2281 agtgtaccat gtgtaaagca aaattggaaa gagaagcaga gagaaaaaat gagtattctc
2341 gctccagatc aaatgggact ggatcagaat cagggaagga tacatgtgat gagtttagaa
2401 gccaaatgaa aaatggaaaa ctcatctgca ctcgagaaag tgaccctgtc cggggtccag
2461 atggcaagac acatggcaat aagtgtacta tgtgtaagga aaaactggaa agggaagcag
2521 ctgaaaaaaa aaagaaagag gatgaagaca ggagcaatac aggagaaagg agcaatacag
2581 gagaaaggag caatgacaaa gaggatctgt gtcgtgaatt tcgaagcatg cagagaaatg
2641 gaaagcttat ctgcaccaga gaaaataacc ctgttcgagg cccatatggc aagatgcaca
2701 tcaataaatg tgctatgtgt cagagcatct ttgatcgaga agctaatgaa agaaaaaaga
2761 aagatgaaga gaaatcaagt agcaagccct caaataatgc aaaggatgag tgcagtgaat
2821 ttcgaaacta tataaggaac aatgaactca tctgccctag agagaatgac ccagtgcacg
2881 gtgctgatgg aaagttctat acaaacaagt gctacatgtg cagagctgtc tttctaacag
2941 aagctttgga aagggcaaag cttcaagaaa agccatccca tgttagagct tctcaagagg
3001 aagacagccc agactctttc agttctctgg attctgagat gtgcaaagac taccgagtat
3061 tgcccaggat aggttatctt tgtccaaagg atttaaagcc tgtctgtggt gacgatggcc
3121 aaacctacaa caatccttgc atgctctgtc atgaaaacct gatacgccaa acaaatacac
3181 acatccgcag tacagggaag tgtgaggaga gcagcacccc aggaaccacc gcagccagca
3241 tgccccccgtc tgacgaatga caggaagatt gttgaaagcc atgagggaaa aaataaaccc
3301 cagttctgaa tcacctacct tcaccatctg tatatacaaa gaattcttcg gagcttgtct
3361 tatttgctat agaaaacaat acagagcttt tgggaatgga ctcactgatt ttcagtcttt
3421 tccatctctt tcctcctaga ctctgtgatc tgagggtata aagacatctc caccaagtct
3481 gagccctcaa aatgtcctga ttacaatgct gtctgtccaa ctgcctgttc aataaaagta
3541 aactcagcag aacacccttt ctgggatttc tttgtcacta tctggataat agatatttgc
3601 ttttaaagaa actgaataaa ctctaccctt ttgtcttttt gtgttgctaa a
