# Supplementary material for: A novel mutation in SPINK5 gene underlies a case of atypical Netherton syndrome
Source: Front Genet. 2022 Sep 9;13:943264. doi: 10.3389/fgene.2022.943264 (PMC9500337; doi:10.3389/fgene.2022.943264)
Supplement: Supplementary file 3 [file DataSheet1.PDF]

Primer	Sequence	Length	Sequencing directio
KRT1-E01-1-F	CAAGCCCAATTTCTTCCCTGTA	570	F
KRT1-E01-1-R	TCTTGTATGCCACCAGGAGGG		
KRT1-E01-2-F	GTGGATTTGGAAGTCGGAGTC	615	F
KRT1-E01-2-R	CCACGAACCCTTTGTCAAAGAG		
KRT1-E02-F	ATCATCTTCTTCCTATTTCTCTGTCA	471	F
KRT1-E02-R	CAAACTGATGTGCCTCTAACCTAAG		
KRT1-E03-F	GAAAGGCAGTGATAGGCAATG	320	F
KRT1-E03-R	CAGTCCAGGTCCATGATGATG		
KRT1-E04+05-F	TTTGCTTGGAGAATCCCCTCA	633	R
KRT1-E04+05-R	GATAGCGTTTGTTAAATGTAGGC		
KRT1-E06-F	AAGCCTAGCCAGTTTCCCTGAA	370	F
KRT1-E06-R	GGAACCAGGGATAATAATGTAGCC		
KRT1-E07-F	GGTTCTGTTGGACTCATTATTGG	446	F
KRT1-E07-R	TCATTTCCCCATACCCAGCA		
KRT1-E08-F	AAGTCTTCAAGGTGGTGGAGC	190	F
KRT1-E08-R	TTCAACCTCAACTCCGTTTCC		
KRT1-E09-F	CTGGAGAATGTGCCCCGAACG	625	F
KRT1-E09-R	ATCGACCTCGGTCTTGCCAAG		
KRT2-E01-1F	TGTACACAACACCTAGTTGGCAGGT	589	F
KRT2-E01-1R	TGACAGAGACTTCGTGGATGC		
KRT2-E01-2F	GGCTTCAGTGGTGGTGGTTTC	373	F
KRT2-E01-2R	ACACCACTGGCTCCTAAGAAATA		
KRT2-E02-F	GCATCCAGCCTCAGACACTTC	424	F
KRT2-E02-R	AACTGGGAGATGAATGGCTTT		
KRT2-E03-F	CATTTGGGGTGAATCCAGTAT	449	R
KRT2-E03-R	CTCTGGCTGCTGCTTTTGC		
KRT2-E04-F	GGGGAGCCTGGGATTTTAGAG	382	F
KRT2-E04-R	TCACCCACAAGACCTATGCTATG		
KRT2-E05-F	GTCTGCGTTTCCCTTATGCTGG	405	F
KRT2-E05-R	GTGCCATTCTCAACATCCTTTCT		
KRT2-E06-F	GACCCTCCCTGGACCAGTAAC	364	F
KRT2-E06-R	ATTTGGGATCGATACATGCTAGA		
KRT2-E07-F	GTTGTTTGCGACTAAACTCCAGA	445	F
KRT2-E07-R	CTCTGCTTTCCCTGTCTTTGC		
KRT2-E08-F	CCCTGGTGGCTGCTTAGAGA	278	R
KRT2-E08-R	GATGAAATGGTGCTGCTTGTC		
KRT2-E09-F	CTGGAGACCTCAGCAGCAATG	619	F
KRT2-E09-R	ACAGAGACAGGCTTCTACATT		
KRT10-E01-1-F	AAGATGCTAAGTTGGCAAAAAG	603	F
KRT10-E01-1-R	CAGGCGGTCATTCAGATTCTG		
KRT10-E01-2-F	TTGGTGGGAGTTATGGAGGCA	457	F
KRT10-E01-2-R	AAGTAACATGGGTAAGCATAGTGAA		
KRT10-E02-F	GGGCACACTTACAGGAATACATCT	447	F
KRT10-E02-R	TGTAAATGTTATTGAGGGCATCC		
KRT10-E03+04-F	AATATTGCCATTACATGAGATCAAC	592	F
KRT10-E03+04-R	AAACAAGGAAAAGGGTGAGGA		
KRT10-E05-F	CAAAGATGCTGAAGCCTGGTT	447	F
KRT10-E05-R	TTTGTTGTATGTTTAATGGCACC		
KRT10-E06-F	CAAATAAGCGTCACCATACTCAAC	369	R
KRT10-E06-R	TGGCATCTTCTTGGGGTTTAG		
KRT10-E07-F	CATTGTTTTCAAAGGATGGGTT	643	F/RS/FS2

KRT10-E07-R	CGGAACCGTCTCTAAGATTTT		
KRT10-E08-F	TGGGCTTAACAATGTAAAGTTGAA	307	F
KRT10-E08-R	CAAGACAGAAGTGTTTTCTTGGAGA		
KRT10-E07-RS	AAACAACTCTAGAGCTTAAAGCGCA		
KRT10-E07-FS2	TGACCTCACCCCGTTTAGTTC		
SPINK5-E25-26-F	GCCTGACTCTTGGAAAGAAA	20	F
SPINK5-E25-26-R	CAGTTGTCACTGGTTCTACA	20	R
